# Experiences with a graduate course on sex and gender medicine in Korea

**DOI:** 10.3352/jeehp.2018.15.13

**Published:** 2018-05-04

**Authors:** Seon Mee Park, Nayoung Kim, Hee Young Paik

**Affiliations:** 1 Department of Internal Medicine, Chungbuk National University Hospital, Chungbuk National University College of Medicine, Cheongju, Korea; 2 Department of Internal Medicine, Seoul National University Bundang Hospital, Seongnam, Korea; 3 Department of Internal Medicine, Seoul National University College of Medicine, Seoul, Korea; 4 Gendered Innovation Center, Seoul, Korea; Hallym University, Korea

Sex and gender medicine (SGM) is defined as the practice of medicine based on the understanding that biology and social roles are important for both men and women in terms of prevention, screening, diagnosis, and treatment [1]. Recent research has demonstrated differences in disease incidence, symptomatology, morbidity, and mortality based on sex and gender [2]. As such, SGM is a fundamental aspect of individualized care. Therefore, the insights yielded by SGM must be considered in medical education and practice, as well as in research. However, much of the scientific evidence about sex or gender differences has not been applied in clinical practice. To improve healthcare outcomes for both women and men, it is essential to understand and apply these differences in clinical care. Integration of sex and gender medical education (SGME) into core medical curricula is essential for achieving competency-based continuing professional development for medical doctors and researchers. Some medical schools in North America and Western European countries have integrated SGME into clinical training programs as part of undergraduate or graduate coursework [3]. Recent experiences with SGME have been reported in the form of reviews, summits, and surveys [4]. According to many participants, SGME is important in clinical practice across various fields of medicine [4]. However, the impact of SGM on clinical practice is limited, primarily because it does not correspond to a specific specialty. To establish SGME as part of the fundamental curriculum, a supporting system, educational materials, structural modules, case studies, and reports about experiences are needed [1,3].

We developed an SGME program as a graduate course, named ‘Sex and gender aspects in biomedical research.’ The present study reports our experiences with SGME and presents an assessment of the impacts of this course on attendees’ knowledge of and attitudes toward SGM. Information from this survey may suggest ways in which sex and gender concepts could be integrated into undergraduate and graduate medical education.

Ethical statement: Informed consent was received from the participants.

This was a comparison study between pre-responses and post-responses after an educational intervention. Twelve students and 10 professors of Seoul National University College of Medicine participated in this study from March 3, 2017 to June 16, 2017. Professors were recruited on a volunteer basis, including 8 professors in clinical medicine, 1 in pharmacology, and the director of ‘Center for Gendered Innovations in Science and Technology Research, Korea Federation of Women’s Science & Technology Associations.’ The professors taught 12 students and participated as subjects of the survey. The 12 students’ majors were medicine (10), community nursing/ nursing system (1), and public health (1). Six of the 10 professors and 8 of the 12 students were male. The course consisted of 15 classes, each lasting from 7:00 PM to 8:30 PM every Friday. The topics included medical diseases organized by organ system and pharmacology. The learning materials were introductory textbooks and additional information on gender-sensitive aspects of diseases, eGender materials, and recent articles. The primary reference was “Sex and gender aspects in clinical medicine,” edited by Oertelt-Prigione and Regitz-Zagrosek [5]. Recent articles were selected by a professor 2 weeks before the class, and all students were notified, including the students assigned to present the content of those articles. The professors made a brief presentation about each topic, including their own research data, followed by the students’ presentation of the article’s contents and a 30-minute analysis. Following this presentation, a comment and discussion session led by the professor and other participants lasted for 30 minutes. The professors met for curriculum planning 1 month before the class. The detailed subjects for each class were planned by professors. The subjects for the 15 classes were as follows: an introductory course, followed by 14 courses on the role of SGM in endocrinology, pulmonary diseases, nephrology, autoimmune diseases, neurology, hematology, cardiovascular disease, psychiatry, hepatology, pancreatic-biliary diseases, the hollow viscera, biomedicine and public health, and pharmacokinetics/pharmacodynamics. At the end of each class, surveys, evaluations, and feedbackgathering from the students were conducted as planned.

Recognition of gender medicine/gendered innovation was surveyed at the beginning and end of the course using the same survey items. The survey tool was adopted from Chin et al. [6] and somewhat modified. The survey instrument consisted of 4 items with a 5-point Likert scale and 2 multiple choice questions (Supplement 1). Pre- and post-class data were compared using the paired t-test to analyze the effects of this class on ideas about SGM. The statistical software used was PASW SPSS ver. 18.0 (SPSS Inc., Chicago, IL, USA).

We compared survey data at baseline and at the end of the course to determine its effects (Table 1). Both teachers and students were initially unfamiliar with sex and gender differences in medicine or the concept of ‘gendered innovation.’ However, they became more familiar with the issues of sex and gender differences in medicine (P< 0.001) and gendered innovation (P< 0.0001) after the course. Most of them agreed that SGM is a fundamental aspect of precision medicine and research (pre- vs. post-class, 81.9% vs. 85.3%; P=0.287) and that SGM should be integrated into routine medical curricula (pre- vs. post-class, 72.7% vs. 86.4%; P= 0.083). In addition, they thought that medical education, research funding, and governmental policies were important for establishing SGM in the biomedical field (Fig. 1). There were no significant differences in the responses between professors and students (P>0.05) or between males and females (P> 0.05). The raw data of this study are shown in Supplement 2.

In the present study, our participants agreed that SGM is a fundamental aspect of precision medicine and research, and that it should be integrated into routine medical curricula. However, they were not familiar with sex and gender differences in medicine or the concept of gendered innovation before the class. Recent studies reported that curriculum gaps in SGBM existed in undergraduate [1] and in graduate [7] medical training in the United States. Most responders agreed that SGM would improve patient management and should be included as part of the medical school curriculum [1]. Despite the importance of this issue, medical education has not adequately integrated SGBM into core curricula. In addition, most of the SGBM material was focused on sex differences in physiology/anatomy and gender differences in disease prevalence, while sex or gender differences in diagnosis, prognosis, treatment, and outcomes were minimally integrated in a medical curriculum [8]. An analysis of graduate medical education also revealed the absence of an instructor or preceptor to discuss the impact of the patient’s sex or gender on patient care during more than half of residency [7]. Students received information regarding sex and gender only when they cared for transgender persons [7]. Therefore, a consensus about the integration of SGM into core medical curricula is needed as soon as possible.

Many reports have revealed that SGM training affected participants’ educational experience favorably in terms of their knowledge, attitudes, and awareness [6]. Therefore, gender-based health issues need to be addressed. In an integrated gender perspective medical curriculum, students learn about the gender effects on health and improve their practical skills to apply gender differences in medical care [9]. Our experience revealed that this course had an impact on participants’ ideas about sex and gender differences in medicine and the concept of gendered innovations.

The barriers to learning more about the impact of sex and gender in medical practice have been found to include limited resources, limited time to learn the entirety of clinical medicine, lack of evidence-based content, and lack of faculty interest. To overcome these barriers, workshops about SGME, international networking [4], elearning materials [10], and search tools have been introduced. We can obtain additional information on gender-sensitive aspects of diseases for SGME from a web-based interactive knowledge-sharing platform [10]. Therefore, to incorporate SGME into the training program, it is crucial to develop teaching materials about SGM-relevant diseases that present clinically significant gender issues.

The study had several limitations. First, there was a small number of subjects, as our sample consisted of the professors of the course itself and a small group of graduate students. Second, we were not able to conduct validity and reliability testing of our survey tool. We adopted a modified version of a previous tool because there was no standard survey tool.

In conclusion, our graduate course on SGME was found to be very effective in changing attitudes toward and knowledge of SGM in both students and professors. We suggest that SGM should be introduced into the curriculum of undergraduate and graduate coursework in medical schools in Korea.

## Figures and Tables

**Fig. 1.**
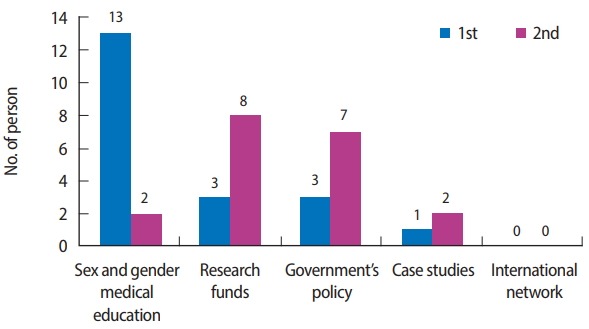
Participant responses to ‘What is the most important factor for establishing gender-based medicine in biomedicine and research (first and second choices)?’.

**Table 1.** Pre- and post-class survey on sex and gender medical education in Korea

Items	Pre-class (n = 22)	Post-class (n = 20)	P-value^a)^
I am familiar with sex and gender differences in medicine.			< 0.0001
Strongly disagree	1 (4.5)	1 (4.5)	
Disagree	12 (54.5)	0	
Neutral	8 (36.4)	8 (36.4)	
Agree	1 (4.5)	9 (40.9)	
Strongly agree	0	2 (9.1)	
I am familiar with the term‘gendered innovation.’			< 0.0001
Strongly disagree	6 (27.3)	1 (4.5)	
Disagree	10 (45.5)	4 (18.2)	
Neutral	6 (27.3)	7 (31.8)	
Agree	0	7 (31.8)	
Strongly agree	0	1 (4.5)	
Sex and gender-based medicine is a fundamental aspect of precision medicine and research.			0.287
Strongly disagree	0	0	
Disagree	1 (4.5)	1 (4.5)	
Neutral	3 (13.6)	0	
Agree	8 (36.4)	7 (31.8)	
Strongly agree	10 (45.5)	12 (54.5)	
Sex and gender issues should be integrated into routine medical curricula.			0.724
Strongly disagree	0	0	
Disagree	1 (4.5)	1 (4.5)	
Neutral	5 (22.7)	0	
Agree	11 (50.0)	9 (40.9)	
Strongly agree	5 (22.7)	10 (45.5)	

Values are presented as number (%).

a) Result of the paired t-test.

## Supplementary material

Supplement 1. Sex and gender medical education survey questionnaire translated into English.

Supplement 2. Data files are available from https://doi.org/10.7910/DVN/73YOBZ.
