# Supplementary material for: Experiences with a graduate course on sex and gender medicine in Korea
Source: J Educ Eval Health Prof. 2018 May 4;15:13. doi: 10.3352/jeehp.2018.15.13 (PMC6004513; doi:10.3352/jeehp.2018.15.13)
Supplement: Supplementary file 1 — Supplement 1. Sex and gender medical education survey questionnaire translated into English. [file jeehp-15-13-suppl.pdf]

Sex and Gender Medical Education Survey

1. Please state the position in this class
- ❑ Student
- ❑ Teacher

2. What is your gender?
- ❑ Male
- ❑ Female

3. What's your final degree?
- ❑ MD
- ❑ PhD
- ❑ Nursing College Degree

4. Please state the timing of survey
- ❑ Pre-class
- ❑ Post-class

Q1 I am familiar with the issue of the sex and gender differences in medicine.
- ❑ Strongly disagree
- ❑ Disagree
- ❑ Neutral
- ❑ Agree
- ❑ Strongly agree

Q2 I am familiar with the term of 'gendered innovation'.
- ❑ Strongly disagree
- ❑ Disagree
- ❑ Neutral
- ❑ Agree
- ❑ Strongly agree

Q3 Sex and gender-based medicine is a fundamental aspect of precision medicine or researches.
- ❑ Strongly disagree
- ❑ Disagree
- ❑ Neutral
- ❑ Agree
- ❑ Strongly agree

Q4 Sex and gender issues should be integrated into routine medical curricula.
- ❑ Strongly disagree
- ❑ Disagree
- ❑ Neutral
- ❑ Agree
- ❑ Strongly agree

Q5 What's the most important to establish gender based medicine in biomedicine and researches? (first and second choice)

First choice

- ❑ Research funds
- ❑ Sex and Gender Medical education
- ❑ Case studies
- ❑ Government’s policy
- ❑ International Network

Second choice

- ❑ Research funds
- ❑ Sex and Gender Medical education
- ❑ Case studies
- ❑ Government’s policy
- ❑ International Network
